# Midline intraprostatic cyst: An unusual cause of lower urinary tract symptoms

**DOI:** 10.4103/0970-1591.38614

**Published:** 2008

**Authors:** Rishi Nayyar, Pankaj Wadhwa, P. N. Dogra

**Affiliations:** Department of Urology, All India Institute of Medical Sciences, New Delhi - 110 029, India

**Keywords:** Midline prostatic cyst, prostatic cyst, transurethral incision, transurethral marsupialization

## Abstract

Symptomatic prostatic cyst presenting as obstructive lower urinary tract symptoms (LUTS) is an infrequent diagnosis in males. Midline cysts are much more likely to obstruct the bladder outlet. We report our experience with four such cases in the last one year, along with a short review of the literature. Two of these cases had additional presenting symptoms besides LUTS - febrile Urinary tract infection (UTI) with perinephric abscess and primary infertility. One case had an anterior midline prostatic cyst which is an extremely rare entity. The remaining three had midline posterior cysts. All cases were treated with transurethral marsupialization, had good relief of symptoms and no adverse effects.

## INTRODUCTION

Prostatic cysts, an erstwhile infrequent diagnosis in males, are usually asymptomatic and mostly detected incidentally during abdominal or trans-rectal ultrasonography (TRUS). Etiological factors include chronic prostatitis as a predominant cause of lateral prostatic cysts and congenital causes for midline cysts. Existent literature on midline prostatic cysts is mostly in the form of isolated case reports, which highlights their uncommon occurrence and even lower propensity for causing symptoms. We report our experience with managing symptomatic benign midline prostatic cysts, including a rare variant of an anterior midline prostatic cyst for which only three cases have been reported so far.

## CASE REPORT

Four young males (Mean age: 28.5 years, Range: 21-35 years) presented to us with lower urinary tract symptoms (LUTS) and/or other associated symptoms [Table 1]. Obstructive LUTS was the primary presenting complaint in all. Case 3 additionally presented with febrile urinary tract infection (UTI) and left perinephric abscess, while Case 4 also had infertility.

**Table 1:** Details of cases with midline prostatic cyst and LUTS

	Case 1	Case 2	Case 3	Case 4
Age (years)	31	27	21	35
Presentation	LUTS	LUTS	LUTS + febrile	LUTS + Primary infertility
			UTI + Lt nephric abscess	
Per rectal examination	Gr 2, firm, smooth, non-tender	Gr 1, firm, smooth, non-tender	Gr 2, firm, smooth, mildly-tender	Gr 2, firm, smooth, non-tender
Location of cyst	Anterior	Posterior	Posterior	Posterior
Peak flow rate (ml/s)	5	7	10	9
Treatment	Endoscopic marsupialization	Endoscopic marsupialization	Endoscopic marsupialization	Endoscopic marsupialization

**Figure 1:**
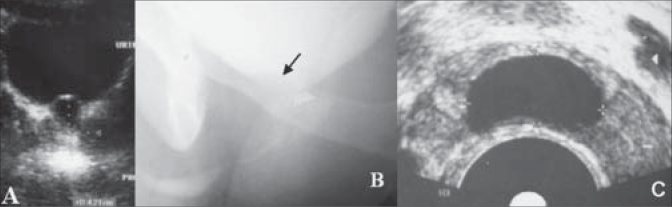
(A) Abdominal ultrasound, (B) VCUG and (C) TRUS image of midline prostatic cyst

Initial evaluation included a urine microscopic analysis and culture, uroflowmetry and a screening abdominal ultrasonography which documented the presence of a prostatic cyst [Figure 1a]. Additional imaging was performed as warranted; TRUS [Figure 1c] was done in all cases, while a contrast enhanced CT scan was performed in one case (Case 3) to evaluate the perinephric abscess. This patient was initially managed with antibiotic therapy and pig-tail drainage of the abscess. Retrograde urethrogram (RGU) and voiding cystourethrogram (VCUG) performed in all cases did not reveal any communication with the prostatic cyst excluding the diagnosis of an enlarged utricular cyst [Figure 1b]. One case (Case 4) was also diagnosed with obstructive azoospermia, unilateral vasal agenesis and absent kidney, in addition to obstructive LUTS. The TRUS revealed a dilated left ejaculatory duct, absent right seminal vesicle and a posterior midline prostatic cyst.

**Figure 2:**
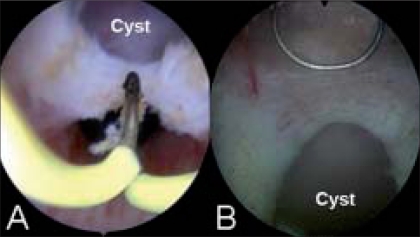
Endoscopic marsupialization of (A) anterior and (B) posterior midline prostatic cyst

All patients underwent cystoscopy and transurethral incision of the prostatic cyst [Figure 2]; Case 4 additionally underwent a transurethral incision of the left ejaculatory duct (TURED). Back pressure changes were noted in all four cases. The roof of the cyst was incised with minimal coagulation under direct vision with a Collins' knife/wire loop to marsupialize the cyst. Care was taken to spare the bladder neck and verumontanum to prevent retrograde ejaculation and incontinence. The incision resulted in drainage of clear fluid. Cold-cup biopsies taken from the cyst wall revealed non-urothelial epithelium with no preneoplastic changes. A 16-F Foley's catheter was placed overnight and patients were discharged the next morning. Follow-up at three and six months demonstrated unobstructed urinary flow and normal ejaculation.

## DISCUSSION

The widespread use of TRUS for evaluating bladder outlet obstruction, infertility, UTI, hemospermia, ejaculatory pain, chronic pelvic pain, prostatic carcinoma or guiding prostatic biopsy has resulted in more frequent identification of prostatic cysts.[1] Most prostatic cysts, however, remain asymptomatic, the symptoms being related to the relative size and position of the cyst. Irrespective of size, the cyst may become symptomatic if it gets infected and also serve as a site for harboring chronic or recurrent UTIs. Midline prostatic cysts have been reported in 1% of men evaluated for LUTS.[2]

Symptomatic prostatic cysts are reported to be associated with prostatic abscess, chronic/recurrent prostatitis; as a cause of chronic pelvic pain, upper or lower UTI, infertility, hemospermia and rarely malignancy.[3,4]

Lower urinary tract symptoms surprisingly are uncommon presenting symptoms, though midline cysts more frequently present with LUTS than laterally placed cysts. The large intraprostatic cyst may compress the prostatic urethral lumen, cause ejaculatory duct obstruction or stretch the prostatic capsule causing pelvic pain.

Etiologically, prostatic cysts include the utricle, mullerian duct cyst, hemorrhagic prostatic cyst, hydatid cyst and cysts associated with prostatitis. Midline cysts, located posteriorly at the prostatic floor, are mostly developmental in origin and arise from remnants of fetal tissue – utricle or müllerian duct. Utricular cysts are endodermal in origin, contain no spermatozoa and are located near the verumontanum, whereas müllerian cysts are mesodermal in origin, contain spermatozoa and are located more posterior and nearer the prostate base. Some cysts are primarily prostatic glandular in origin and are acquired later in life. Most lateral prostatic cysts are related to chronic prostatitis, where they may play a causative role or may be consequent to it. Anterior midline cysts of prostatic origin are extremely rare with only three cases reported in the literature so far.[5–7] Case 1 in our series who presented with bladder outlet obstruction also had such an anterior midline prostatic cyst.

In some cases it may be fairly easy to directly relate the presence of the prostatic cyst as the etiology behind the patient's clinical symptoms. Though the diagnosis of a midline prostatic cyst can be made on TRUS examination, the functional implication of the midline prostatic cyst cannot be determined by TRUS alone.[8] Thus, in patients with urologic symptoms, detection of midline cysts will require a focused examination to determine whether the cysts represent a normal variant or are the cause of the symptoms. Further studies are needed to determine which imaging or clinical characteristics may be useful to distinguish the normal variant cyst from the cyst that is responsible for urologic symptoms.

Various therapeutic options for managing midline prostatic cysts described include transrectal aspiration with or without sclerotherapy, transurethral marsupialization and open surgery.[9] Dik *et al.*, reported durable, recurrence-free results in a series of patients with medial prostatic cyst treated with transurethral incision.[3] Similarly, Chang *et al.*, reported excellent outcome with transurethral resection of midline prostatic cyst projecting into the prostatic urethra and presenting as obstructive LUTS.[10]
